# Evolving Social Dimension of the European Higher Education Area

**DOI:** 10.1007/978-3-030-56316-5_11

**Published:** 2020-07-26

**Authors:** David Crosier, Cezar Mihai Haj

**Affiliations:** 1 UNESCO Chair on Science and Innovation Policies, National University of Political Studies and Public Administration, Bucharest, Romania; 2 European Commission, Brussels, Belgium; 3 National University of Political Studies and Public Administration, Bucharest, Romania; 4 Eurydice, Brussels, Belgium; 5 Executive Agency for Higher Education, Research, Development and Innovation Funding, Bucharest, Romania

## Abstract

The social dimension is a term coined in the early years of the Bologna Process (BP). Although mentioned in the early ministerial communiqué texts, the term itself was not clearly defined until 2007. Looking back, this could perhaps be considered an oversight. However, the more likely explanation is that the term was chosen intentionally, leaving open possibilities for national and institutional action while, at the same time, committing countries to nothing precise.

## The Social Dimension in the Bologna Process

...student body entering, participating in and completing higher education at all levels should reflect the diversity of our populations’ (Bologna Process Bologna Process 2007a)

The social dimension is a term coined in the early years of the Bologna Process (BP). Although mentioned in the early ministerial communiqué texts, the term itself was not clearly defined until 2007. Looking back, this could perhaps be considered an oversight. However, the more likely explanation is that the term was chosen intentionally, leaving open possibilities for national and institutional action while, at the same time, committing countries to nothing precise. In the early years of policy-setting for the European Higher Education Area (EHEA), the use of the term “dimension” was indeed a signal of aspiration in a rather loosely defined policy area. Thus, as well as the “social dimension”, references can be found in Bologna documents (communiqués and working group reports) to the “European dimension” of higher education as well as to the “international dimension” and also to the “external dimension” of the Bologna process. None of these terms was accompanied by clear definitions.

An important moment for the social dimension in the Bologna Process came in 2007 when in London the higher education ministers adopted the recommended definition of the working group that had been mandated to reflect on how policy in this area should be developed. They defined the objective of social dimension policy to be that the ‘*student body entering, participating in and completing higher education at all levels should reflect the diversity of our populations*’ (Bologna Process 2007a). This definition continues to be a valid and actual reference point. It is a definition that embraces a clear, aspirational objective—the representation of the diversity of the population. It also entails a process to reach the goal—i.e. the development and implementation of policies and practices (Bologna Process 2007b). Most importantly, establishing this definition brought clarity to discussions.

Everything that has been written in subsequent communiqués and EHEA policy texts derives from and sometimes repeats the 2007 Working Group report and the definition in the London Communiqué. Thus “*Student body within higher education should reflect the diversity of Europe’s populations* (Bologna Process 2009)”,“*The student body entering and graduating from higher education institutions should reflect the diversity of Europe’s populations* (Bologna Process 2012)”, “....*the student body entering and graduating from European higher education institutions should reflect the diversity of Europe’s populations*...(Bologna Process 2018)”

Some subsequent communiqués focus on commitment to develop and implement national strategies (Bologna Process 2009, 2012) while others give particular emphasis to other aspects. For example, the higher education role in building inclusive societies is a focus of the 2015 Yerevan Communiqué. In this document, ministers agree to ‘*enhance the social dimension of higher education, improve gender balance and widen opportunities for access and completion, including international mobility, for students from disadvantaged backgrounds*.’ (Bologna Process 2015). The communique further emphasises flexible learning paths and the inclusion of different types of learners by promoting life-long learning and recognition of prior learning.

While responsibility lies with countries and institutions, there have nevertheless been important developments in European-level discussions throughout the Bologna period. Despite each country being left to determine the particular parameters of under-representation in its specific context, a European discussion has required some broad categories of under-represented groups to be identified. These categories may also correspond to notions of disadvantaged or vulnerable students in national contexts and commonly include people from a low socio-economic background (identified through low income or low educational background of parents), gender, disability and immigrant or minority status. Mature students are also often included. European discussions also recognise that students from particular geographical regions may be disadvantaged—particularly those in isolated rural areas or areas of high urban social deprivation, and that under-representation is also associated with insufficient formal education qualifications for entry into higher education.

These categories of under-represented, vulnerable or disadvantaged groups are not separate, discreet entities. In lived reality, they combine as characteristics of individual citizens, and under-representation may be apparent in a combination of these different factors. Thus, a person from a low socio-economic status background will also have a gender, may come from an area of social deprivation, may be a migrant or have a disability, and all of these factors will combine to play a role in shaping or limiting opportunities in a particular context. Within the Bologna Process, the main discussion focuses on the need to develop strategies and actions to improve access to, participation in and completion of higher education for members of these groups.

This paper aims to present an overview of the ways in which the issues under the umbrella term of “social dimension” have been conceptualised and addressed by higher education policy throughout the Bologna Process. It also considers the challenges that lie ahead in improving social reality in European higher education and introduces the papers that have been selected to provide in-depth research on different aspects of the social dimension.

## Social Dimension Strategy

Despite discussion and commitment for national strategies and action plans (Bologna Process 2007a), there are few countries that have actually made a conscious effort to develop genuine social dimension strategies in the European higher education Area. Ireland is perhaps the most notable exception to this general rule. Here, a succession of higher education policy strategies has consistently featured inclusion and widening participation as high-level policy goals throughout the Bologna period. The country has also developed support measures focused at students from under-represented groups—particularly mature students, students from the traveller community and students with disabilities. Ireland has also established good quality monitoring systems to track the impact of policy—measuring completion and drop-out rates for specific under-represented groups. (Department of Education and Skills, Dublin 2011)

The focus on social inclusion has not inhibited other policy initiatives. On the contrary, Ireland’s strategy has engaged fully with other trends in learning and teaching, research and innovation, community engagement and internationalisation. Indeed, the country’s approach offers a good model for the wider EHEA commitment being prepared for the 2020 Rome Communiqué to make the social dimension a core commitment on which to build all future policy.

Other countries also offer interesting models. In particular, the Nordic countries demonstrate a model of social equity that permeates higher education. This can be most clearly seen through the type of financial measures that are used to support students. In contrast to nearly all other European systems, no tuition fees are charged to national and European students. This policy also extends in some countries to all international students, but in recent years, countries such as Denmark and Sweden have introduced fees for students from outside Europe. (European Commission/EACEA/Eurydice 2018)

In addition to the absence of fees, a very high proportion of students receive grants, with amounts usually adjusted according to the individual student’s socio-economic situation. The public investment is, therefore, considerable and provided in a mainstream form.

One important aspect of this Nordic model is that no specific groups are identified. Through providing generous conditions and support to all students, disadvantaged or vulnerable students also receive sufficient support and are not stigmatised.

While one major benefit of this model is its inherent inclusivity, its most significant disadvantage is that there is no way of knowing how many students from under-represented, disadvantaged or vulnerable groups actually benefit from the support. As the notion of groups is anathema to a system designed to support everyone, there remains a potential for hidden inequity to continue without specific action being taken to address it.

Almost all other European higher education systems take a different approach to student financing. Most commonly, countries charge tuition fees to some students—but provide fee reductions or exemptions to students who are considered as ‘disadvantaged’. This refers to students with low socio-economic background and those belonging to various under-represented groups, such as students with disabilities, ethnic minorities, orphans or, in a few countries, children of war victims. Fee waivers or reductions based on socio-economic criteria generally concern a relatively small proportion of students.

A number of transition countries such as Latvia, Lithuania, Hungary, Romania, Bosnia and Herzegovina, Serbia and North Macedonia use merit-based criteria in differentiating fees paid by full-time higher education entrants. In these countries, based on performance in upper secondary education and/or results of admission tests, students start their higher education studies in one of two groups: a ‘state-funded’ group, including better-performing students who do not pay fees or pay only small administrative charges, and a group of ‘self-financing’ students who cover (fully or partially) their tuition expenses. Depending on the country, self-financing students may comprise between 30% and 60% of students.

Even more countries use merit-based criteria for allocating grants. According to Eurydice data, in 22 higher education systems, grants are awarded either based on educational outcomes during higher education studies or based on secondary school results or performance in admission tests.

This merit-based approach to student funding appears to be blind to the reality that it rewards previous social and educational advantage. Yet research consistently shows that students coming from families where parents have themselves benefitted from higher education have greater opportunity to access higher education. Providing fee waivers and grants on the basis of merit-based criteria thus shores up social advantage and inequity.

One of the reasons why merit-based student funding may not be a high profile social justice concern for citizens or policy makers is that almost all countries also have parallel support that is attributed on a needs basis. Indeed, Eurydice shows that in 32 higher education systems, grants exist that prioritise socially or economically disadvantaged students.

While student financing is widely understood as an important aspect of the social dimension, there is little evidence that specific funding approaches are effective in widening participation. The United Kingdom provides an interesting case study in this respect, as England (and to a lesser extent Wales and Northern Ireland) operates a system where high fees (capped at £9 250/€10 000 per year) are charged to all students. However, these fees are only paid after the student graduates, and when she or he is earning over a specific income threshold. In combination with these fees, students may take out loans which also have to be repaid after graduation. Most prospective students, therefore, begin their study programmes in the knowledge that they will accrue high levels of debt.

While this system transfers some of the cost of higher education from state to individuals who participate, it has also been designed with features to support under-represented groups. Indeed, higher education institutions have to sign access agreements that aim to ensure equality of opportunity for students from low-income and other under-represented groups and to increase retention rates. Some of the fee funding is thus redistributed to low income and other under-represented groups through specific measures at institutional level, while higher education institutions also take other action in terms of service provision to support disadvantaged students.

Scotland, which has full responsibility for its education system, takes a radically different approach to student funding. No fees are charged in short and first cycle higher education to Scottish and European students—except to those from the rest of the United Kingdom. Meanwhile, a combination of grants and loans support students from disadvantaged backgrounds.

Interestingly, there is no clear evidence that one approach is having a more positive impact than the other on widening participation to students from under-represented groups. The evidence suggests rather that the factors affecting participation of under-represented and disadvantaged groups run deeper than funding. In other words, if funding at higher education level is the main mechanism used to attempt to widen participation, then success is likely to be limited.

Austria and Romania are both countries which have made attempts to take a broader strategic approach to social dimension issues in recent years. These countries are thus the subject of a comparison of experience in the paper by Torotcoi. Austria has established long-term targets for increasing participation from under-represented groups and has set multiple goals for different societal groups. (BMWFW 2017) Stakeholder representatives have all been involved in defining and developing the objectives, and there appears to be a widespread understanding of the underlying rationale for action. Torotcoi demonstrates, however, that other national strategies and policies are in contradiction with the objectives of higher education social dimension strategy, and hence the likelihood of deeply-rooted change is diminished.

In contrast, Romanian universities have worked individually to define their social dimension strategy and have then been brought together through stakeholder interaction. However, so far improving the social dimension has not proved to be a sufficiently strong motivating factor for universities to form a true critical mass. A broader debate and greater societal engagement will be needed if there is to be agreement on the direction of such social inclusion policies. This debate needs to engage actors in social policy across a number of sectors and involve different responsible government ministries.

## Exploring Social Dimension Policies

One of the main challenges for research in this field is that it has often been limited to exploration of policy measures taken at higher education level. There are many rational reasons for this research choice. Among them is the fact that policy-making is often differentiated by sectors in national systems. While some governments have a ministry responsible for all aspects of the education system, many countries separate ministerial responsibility for school and higher education. Moreover, even in more unified systems, administrative divisions may facilitate separate spheres for action in higher education.

One important study (Orr et al. 2017) examined the impact of admission systems on higher education outcomes—including opportunities for access as well as successful completion of degree programmes. This study showed that admission is best conceptualised as a process that is developed through the relationship between different parts of the education system. Thus in some countries, the fact that a significant share of lower secondary education pupils may be directed into educational paths that do not provide access to higher education is a stage of defining who can and cannot be admitted to higher education. The implication is that, if the social dimension agenda is to be coherent, it must consider how this kind of system feature can be reformed to ensure that equity runs throughout the education system. This approach would apply from early childhood education and care, through primary and secondary education as well as higher education.

Gender is another aspect of the social dimension that illustrates the difficulty of taking action only at higher education level. The 2018 Bologna Process Implementation Report shows that fields of study are highly differentiated by gender. Women make up almost 80% of first cycle students enrolled in subjects related to education, health and welfare, but less than 20% of the students in information and communication technologies. (European Commission/EACEA/Eurydice 2018)

This reality is shaped by our societies from early childhood education onwards. Without awareness of gender differentiation and action to address it coherently throughout society, there is little hope that the pattern of participation in higher education programmes would change. Indeed, it is a moot point whether this is an issue that can be significantly affected by higher education policy reform.

Higher education is also a sector that often seems to be more reactive than proactive in the face of certain societal developments. One example is demographic change. Since 2010, many EHEA systems, particularly Ukraine, Kazakhstan, Estonia, Poland, Romania, Slovenia, Slovakia and Lithuania, have been experiencing a decline in the numbers of secondary school leaving students seeking access to higher education. (European Commission/EACEA/Eurydice 2018)

This demographic decline could have been a catalyst for policy makers and higher education institutions to innovate in adjusting their offer. Such innovation could, for example, have comprised the exploration of targeting new potential learners in higher education. Admission through the recognition of prior learning, or other criteria than achievement of upper secondary education could have been explored. Alternatively, countries could have invested in preparatory programmes to develop a baseline of knowledge, skills and competences in more mature returners to higher education.

It is noticeable, however, that none of the countries mentioned above has taken steps to make this kind of adaptations to their system. (European Commission/EACEA/Eurydice 2018) Rather, they remain steadfast in the model of higher education on offer. While there may be discussion to broaden access among the typical higher education age group, there has been little or no reflection on changing the nature of the programme offer.

Two of the papers in this section consider the way in which social dimension policy is having an impact on under-represented groups in different parts of Europe. Torotcoi et al. explore the question of whether existing policy measures have demonstrated success in increasing participation of under-represented groups. Through analysis of research in the field, it becomes clear that this question has no simple answer. Just as different characteristics combine in the single identity of any given citizen, so too do different factors affect the impact of actions and measures in any particular context. Guidance and counselling services, for example, may be highly effective to support certain students in accessing higher education and studying. However, the quality of these services, their ability to identify and target students in need of support and myriad other factors may affect their impact. Similarly, funding support to disadvantaged students can intuitively be recognised as a necessary measure to support those students with few financial resources to participate in higher education. However, if this is an isolated policy action that is not complemented by other elements of a widening participation strategy, then it is very unlikely to stimulate significant change. A further problem identified in this paper is that policies may often be developed without sufficient attention to the monitoring required to assess their impact.

The outcomes of this work emphasise the complexity of understanding reality, of developing effective policy, and of assessing impact. The research confirms the enormous need for quality data and research to improve understanding of social reality and to develop and refine more effective policy interventions.

Brooks et al. approach the issue of policy needs in this area from the perspective of learners and staff. The research explores how staff and learners perceive the impact of social characteristics on higher education experience. They do this through a research project focused on subjective perception of staff and learners’ experience of social characteristics in seven European countries. The research uses focus group discussions to reveal a number of interesting findings. Notable among them is that staff and student perceptions of social reality differ significantly. While staff may be conscious of certain characteristics in the student population—particularly characteristics often featured in national policy and media discussion—they are often seemingly unaware of others. Students, on the other hand, appear to have a higher level of awareness of the different social characteristics of their peers, as well as of the impact that these characteristics may have. Discussions with students thus tended to consider a broader range of factors than those with staff. The research also revealed significant national differences in the perception of impact of social characteristics on opportunities to access higher education. In particular, in Poland—which was the only example of a post-soviet transition country in the sample—there was a widespread view that there is no issue of under-representation to be tackled.

These findings give further arguments to pursue some of the challenges that the European Higher Education Area faces. Firstly, there is clearly a need to engage students in policy discussions—particularly in relation to social dimension issues if these are to be tackled effectively. Secondly, it is essential to provide training to staff to raise awareness of how social characteristics may affect student learning and the overall higher education experience. There is also a need to improve understanding of how education provision could better respond to a broader range of needs in a more diverse student body. Finally, it will be important to assess the impact of measures taken to address these challenges. Effective monitoring, therefore, needs to be built in to all policy actions.

## Lack of Impact of the Bologna Process in the Social Dimension

Although the social dimension has remained a constant discussion on the Bologna Process agenda, it is difficult to find evidence that the process itself has been an effective vehicle for addressing social dimension challenges. Nevertheless, the 2018 Bologna Process Implementation Report shows that most countries have some measures in place designed to improve inclusion. As already discussed, financial support for students from under-represented groups exists almost everywhere, and access in some systems has also been facilitated by preferential treatment and opening alternative entry routes. Outreach programmes and information campaigns directed for under-represented groups are also commonly used, and counselling and guidance services are widespread. Performance indicators for institutions often now include financial incentives for institutions to improve access and completion for under-represented groups. However, there is no macro-level correlation of the existence of these measures with any improved outcomes.

The main aspects of inequity have remained constant throughout the Bologna period and across geographical boundaries. Under-representation persists in all countries, despite a variety in policies and diversity in the underlying socio- economic conditions of countries. There remains a clear correlation between high educational background of parents and the chance of their children entering higher education. (European Commission/EACEA/Eurydice 2018)

Gender differences in certain study subjects persist and are strengthening. Students with an immigrant background are also clearly under-represented in most EHEA higher education systems. Meanwhile, although the numbers of mature students vary between countries, life-long learning is often not a significant reality. Few inroads have been made to any of these aspects of reality.

## Monitoring: Neglect in Social Dimension Policy

One important aspect of social dimension policy is that impact—positive or otherwise—can only be assessed if policy is underpinned by effective monitoring. All countries monitor the composition of the student body to some extent. However, when monitoring practice is examined more closely, gaps are revealed.

The 2018 Bologna Process Implementation Report shows that EHEA countries report that the student body is monitored usually at entry and during studies, but to a lesser degree at graduation and least often after graduation. This means that, even if information on the social composition of the student body can be provided at entry and during studies, comparable information is not collected at the point of graduation, and thus the effectiveness of measures and services in supporting under-represented groups through higher education is impossible to determine. Even less is known about what happens to graduates beyond higher education and whether social factors continue to have an impact on their opportunities in the labour market.

The lack of systematic monitoring at key stages of higher education is compounded by two other issues. The first is that monitoring tends to be undertaken as a snapshot—revealing a picture of current reality. It is rare to find systems that monitor the development of cohorts or track individual students. This type of tracking, however, is needed to allow a clearer picture to emerge in relation to the particular characteristics of the student population.

The second problem is that, even when administrative monitoring is undertaken, many relevant social characteristics are commonly overlooked. Across the EHEA, it is unsurprising to find that gender and age are routinely monitored. Beyond that, the picture varies. Many systems collect data on disability, but this is likely to be far from comprehensive as many students with impairments may choose not to disclose them for fear of stigma. (e.g. Eurostudent) Data on socio-economic background is also often collected in relation to student support systems. However, it may not be used for policy reflection. Data on migrant or ethnic minority status is much less often gathered. Overall, according to the 2018 Bologna Process Implementation Report, only 11 EHEA countries have reliable data on completion rates of studies for students from under-represented or disadvantaged groups. The other 37 countries, despite monitoring overall completion and drop-out rates, do not collect information in relation to specific groups.

The only conclusion that can be drawn from this information is that this rather ineffectual action in monitoring means that social dimension policy implementation cannot be considered as a great priority. If it were, there would be available data gathering systems in place to understand the impact of policy.

## New Social Dimension Challenges

The issues already outlined in this paper provide a massive policy agenda implying, as they do, a major effort at holistic education system reform. Nevertheless, the reality that we face today is fast-moving and rapidly changing, as exemplified by the sudden onset of the covid-19 pandemic. The closing of higher education institutions is being accompanied by a surge in online provision, and this temporary reality will no doubt drive a shift in attitudes towards digitalisation that will have some permanent repercussions. The social dimension, therefore, needs to take account of the changes in the global context as well as the potential opportunities provided by new developments.

The social dimension is inextricably related to sustainable development, another paradigm that must guide future policy-making. The objective must be to harness new technologies, as well as new ways of thinking and collaborating to face the challenges in our world. This is the logic that has been followed within the Bologna Process by moving the social dimension to a core commitment for future development. It implies that other emerging challenges—such as the harnessing of digital technology to support inclusive teaching and research, or the integration of micro-credentials into the higher education framework—are understood as part of a new social dimension paradigm.

## The Integration of Refugees and Asylum Seekers

From 2015, Europe was faced with what at the time was often described as a “migration crisis”. This followed the significant increase in numbers of asylum seekers and refugees seeking to enter European Union countries as a result of war in Syria, and continued post-conflict societal degeneration in a number of other states—with the most significant refugee numbers coming from Afghanistan.

Mass migration is not a new phenomenon: while the factors behind mass movements of people change, the reality of migration has been with us throughout the modern era. So while the increase in numbers of asylum seekers to European shores was an unusual and unexpected event, it is inevitable that migration will continue to be a feature of our lives in the future. We may not be able to predict the precise causes of future mass migration, but we can prepare for such events knowing that they will occur at some point.

Two of the papers in this volume—by Berg and Erdogan—consider the emergence of refugees and asylum seekers as a group affecting the social dynamics of higher education systems in recent years. While media reporting tended to focus attention on the potential impact of asylum seekers and refugees in each individual country, in reality, the only European Union countries that responded through opening up their countries to refugees to any significant extent were Germany and Sweden.

It was the neighbouring countries to Syria that accepted the vast majority of asylum seekers and refugees. Foremost among these countries is Turkey, which in 2020 has a population of more than 4 million asylum seekers and refugees—the largest population of displaced people in the world. Overall, among all Syrian asylum seekers fleeing the country in 2015 and 2016, more than 50% were in the age category 18–34. Among these, many have been seeking to develop their educational skills since leaving Syria. This provides challenges as well as opportunities for the host country in developing skilled and educated citizens.

The paper from Erdogan is based upon survey research with Syrian refugees studying in the Turkish higher education system. The research explores a variety of aspects of the refugees’ experience of Turkish higher education, and the findings provide an important overview of the extent to which this vulnerable population has been supported into higher education institutions. The findings are relevant for improving Turkish action and measures. However, they are equally relevant for wider European policy-thinking—especially if European countries are to prepare better to integrate asylum seekers and refugees in the future. It is, of course, impossible to predict when a significant new wave of refugees will be in need of asylum nor in relation to what particular conflict or natural disaster. However, it is entirely possible to predict, on the basis of past experience and known realities about climate emergency, that the future will see significant numbers of new refugees arriving at some point. It would, therefore, be sensible to prepare for such an eventuality.

Berg consider the integration of Syrian refugees into Germany—the European Union country that also welcomed a large number of refugees in 2015 and 2016. The research explores the transition into higher education, focusing on refugee students, and highlighting the ways in which the experience for refugees may differ from ideal or typical transitions. The paper illustrates how professionals working in the higher education system may need to think carefully about their routine practice. While “typical” native students may have acquired knowledge of the transition process, many of these aspects cannot be assumed for refugee students. Hence there is a need to think ahead of potential barriers and the support that refugees may need. To do this effectively, it is important to find out directly from refugee students how they experienced the process.

This research focuses on how often the provision of information is constructed with a particular idea of a student (coming out of secondary education) in mind, and not considering the needs of vulnerable students. It also shows how counselling services may carry unconscious or conscious biases that mean that sometimes refugee students are not encouraged to develop their full potential but rather to enter low- level education programmes.

Both papers on refugee students point to the need not only for the potential student to adapt to the higher education system, but also for the system, institution and support services to adapt to the needs of the student. The capacity of a system to do this effectively should be considered as a measure of success in the social dimension.

## The Third Cycle

Perhaps because only a small minority of students studies in third cycle programmes, these studies have often been neglected in considerations of the social dimension. The research undertaken by O’Regan addresses this cycle, focusing in particular on differences between full and part-time doctoral candidates in Ireland in access to programme-based information and academic and personal support networks.

In the particular sample selected, part-time candidates have a higher share of mature, female students. The findings suggest that awareness and consideration of the needs of this group of students would enable information and support to be more effectively delivered. While this research draws on information related to a specific geo-cultural reality in Irish higher education, the outcomes are worthy of more general reflection. Indeed, they reinforce points revealed in the research by Brooks et al. and by Berg that academic staff often appear not to be sufficiently aware of students’ social characteristics, or of the impact that these characteristics may have on their learning experience.

## Conclusions: Addressing the Challenges Ahead

The social dimension, despite being a broad concept, has been clearly defined in the European Higher Education Area since 2007. The definition of the London Communiqué responds to a basic question for citizens and policy makers alike, “what kind of higher education system do we wish to develop?” The response contained in the definition is that we are striving for a higher education that is open and equitable, and where the diversity of our populations is reflected in higher education.

Although the goal has been acknowledged throughout the Bologna Process, it has largely remained an aspiration. No country has achieved the objective, and most countries have taken little action even to move towards it. Indeed, the social dimension has remained a largely peripheral concern of the EHEA, with periodic reminders in subsequent communiqués to develop strategies to address the topic.

While the social dimension has been largely a neglected aspect of EHEA attention, there are now signs that this is set to change. Recent work undertaken by the BFUG Advisory Group for the social dimension has produced a document that aims to push the social dimension agenda to the forefront of policy discussion in the Bologna Process. The Principles and Guidelines to Strengthen the Social Dimension of Higher Education in the EHEA will be a highly beneficial tool for any government willing to address this area seriously. They provide a clear and concise set of issues that require reflection and debate (Bologna Process 2020).

The Principles and Guidelines document establishes ten principles, accompanying each principle with guidelines on how they could be implemented. It should be adopted by Ministers in Rome, November 2020, alongside a strong statement on why the social dimension should be at the core of the Bologna Process.

Another reason to hope that the Principles and Guidelines may stimulate positive change is that it is a very comprehensive overview of the issues that need to be considered. It provides the perspective of long-term commitment, with governments mainstreaming social dimension, accessibility, equity and inclusion in all aspects of their education systems. If implemented seriously, it may act as a catalyst for the type of holistic thinking around education systems that is essential for social dimension challenges really to be addressed.

The Principles and Guidelines also consider the main areas of action required from higher education institutions and the fact that they need support to strengthen their capacity to respond to the needs of a more diverse student body. The document highlights the need for effective counselling and guidance and raises questions about the type of student funding required to strengthen the social dimension. These actions will require good systems for monitoring, and this task is also fully incorporated within the Principles and Guidelines.

The social dimension clearly requires major policy attention. European higher education serves many purposes, but until now, it is not a strong force in redressing societal inequity. Indeed, admissions systems, curriculum organisation and resource distribution are currently more likely to contribute to and strengthen inequity than to tackle it. Greater understanding of social reality is clearly needed, and better data gathering systems must be developed. However, it would be wrong to wait for better data before taking policy action. The process of addressing social dimension challenges needs to begin now as a matter of urgency.
